# First hospital outbreak of the globally emerging *Candida auris* in a European hospital

**DOI:** 10.1186/s13756-016-0132-5

**Published:** 2016-10-19

**Authors:** Silke Schelenz, Ferry Hagen, Johanna L. Rhodes, Alireza Abdolrasouli, Anuradha Chowdhary, Anne Hall, Lisa Ryan, Joanne Shackleton, Richard Trimlett, Jacques F. Meis, Darius Armstrong-James, Matthew C. Fisher

**Affiliations:** 1 Department of Microbiology, Royal Brompton Hospital, London, UK; 2 Department of Medical Microbiology and Infectious Diseases, Canisius Wilhelmina Hospital (CWZ), Nijmegen, The Netherlands; 3 Department of Infectious Disease Epidemiology, Imperial College School of Public Health, St Mary’s Campus, London, UK; 4 Department of Medical Mycology, Vallabhbhai Patel Chest Institute, University of Delhi, Delhi, India; 5 Department of Surgery, Royal Brompton Hospital, London, UK; 6 Radboudumc/CWZ Centre of Expertise in Mycology, Nijmegen, The Netherlands

**Keywords:** *Candida auris*, Outbreak, Healthcare-associated infections, AFLP Genotyping

## Abstract

**Background:**

*Candida auris* is a globally emerging multidrug resistant fungal pathogen causing nosocomial transmission. We report an ongoing outbreak of *C. auris* in a London cardio-thoracic center between April 2015 and July 2016. This is the first report of *C. auris* in Europe and the largest outbreak so far. We describe the identification, investigation and implementation of control measures.

**Methods:**

Data on *C. auris* case demographics, environmental screening, implementation of infection prevention/control measures, and antifungal susceptibility of patient isolates were prospectively recorded then analysed retrospectively. Speciation of *C. aur*is was performed by MALDI-TOF and typing of outbreak isolates performed by amplified fragment length polymorphism (AFLP).

**Results:**

This report describes an ongoing outbreak of 50 *C. auris* cases over the first 16 month (April 2015 to July 2016) within a single Hospital Trust in London. A total of 44 % (*n* = 22/50) patients developed possible or proven *C. auris* infection with a candidaemia rate of 18 % (*n* = 9/50). Environmental sampling showed persistent presence of the yeast around bed space areas. Implementation of strict infection and prevention control measures included: isolation of cases and their contacts, wearing of personal protective clothing by health care workers, screening of patients on affected wards, skin decontamination with chlorhexidine, environmental cleaning with chorine based reagents and hydrogen peroxide vapour. Genotyping with AFLP demonstrated that *C. auris* isolates from the same geographic region clustered.

**Conclusion:**

This ongoing outbreak with genotypically closely related *C. auris* highlights the importance of appropriate species identification and rapid detection of cases in order to contain hospital acquired transmission.

## Background

The fungal pathogen *Candida auris* (*C.auris*) was first described in 2009 after isolation from the ear of a patient in Japan, and is responsible for a wide range of healthcare associated invasive infections [1]. Since its first description, reports of nosocomial outbreaks of *C. auris* have been reported from India [2], South Korea [3], South Africa [4] and Venezuela [5]. *C. auris* is phylogenetically related to *C. haemulonii* and accurate species identification is necessary for the administration of appropriate antifungal therapy and the control of outbreaks in hospital settings [6].

## Methods

### Outbreak setting and epidemiological data

The outbreak involves the Royal Brompton Hospital in London (United Kingdom) which is a National Health specialist centre for cardio-thoracic surgery with 296 beds. *C. auris* cases were identified by routine microbiology cultures from clinical sites such as wound swabs, urine samples, vascular devices tips, blood cultures as well as skin screening samples (including nose, axilla, groin and stool samples) of patients exposed to *C. auris* cases or an environment where positive patients were previously based. Demographics including gender, age and ward were collected prospectively. All microbiological samples were prospectively collected and data retrieved using the laboratory Information Management system (WinPath v5.32 software, Clinisys Solutions Ltd, Chertsey, UK).

### Definitions

Colonization with *C. auris* was defined as culture positive skin, oropharynx, vascular line exit site, respiratory, and urinary tract without clinical signs of *Candida* infection. Candidaemia episode was defined as a *Candida* positive blood culture (BC) treated within a three months period. Possible *C. auris* infection was defined as a case with a positive culture from a non-sterile site (sternal wound, urine, vascular line tip) and clinical signs and symptoms of infection requiring treatment with antifungal agents. Presumed invasive candidiasis of unknown focus of infection was defined as a patient demonstrating raised inflammatory markers despite the use of broad-spectrum antibiotics and responding to systemic antifungal treatment and/or expressing additionally raised serum β-D-glucan (BDG).

### Laboratory methods

*Candida* isolates from clinical, patient and staff screening and environmental swabs were plated on Sabouraud dextrose agar plates and identified using Chromogenic ager (Brilliance *Candida* Agar, Thermo Scientific, Basingstoke, UK). Non-*C.albicans* isolates including *C. auris* were speciated by Matrix Assisted Laser Desorption Ionization-Time of Flight mass spectrometry (MALDI-TOF; Bruker, Bremen, Germany) using the Biotyper v3.1 software (Bruker Ltd, Coventry, UK). Antifungal susceptibility testing was done by microbroth dilution (Sensititre YeastOne; Trek Diagnostic Systems Ltd, East Grinstead, UK).

Typing of *Candida* isolates from representative isolates of a number of global outbreaks was done by AFLP analysis as previously described [7, 8]. Briefly, genomic DNA was extracted from 48 h liquid cultures using the MasterPure yeast DNA purification kit (Epicentre Biotechnologies, Cambridge, United Kingdom) with an additional bead beating step included. Extracted gDNA was quantified using a Qubit 2.0 fluorometer and dsDNA BR (double-stranded DNA, broad range) assay kit (Life Technologies, Carlsbad, CA, USA).

## Results

### Outbreak description

We report an ongoing outbreak describing the first 16 month experience. During this period there were a total of 50 *C. auris* cases (17 female, 33 male; average age 53 years, range 19-78) within a single Hospital Trust in London. In April 2015 the first patient was identified in a 20 bedded mixed medical-surgical adult intensive care unit (ICU) of a specialist cardio-thoracic centre. The yeast was initially cultured from a sternal wound. Within one week a second patient in the adjacent bed to the index case became *C. auris* culture positive in their sputum and subsequently developed an intravascular line related infection. Both patients had undergone cardiac surgery and required treatment with the antifungal agent caspofungin for their infection. A 12-month retrospective microbiology database search showed that *C. auris* had not been previously isolated from any patient. At the time it was thought that hospital acquired transmission from a single index case was likely but no obvious source was apparent.

**Fig. 1:**
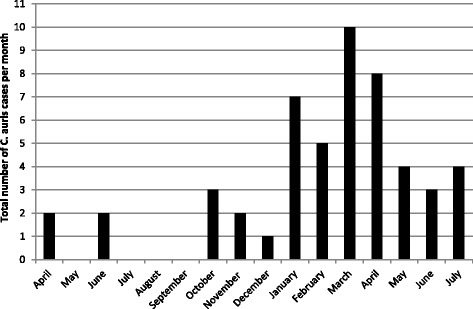
New cases of *C. auris* per month. Total number of monthly new cases of *C. auris* are listed from the 1 April 2015 to the end of July 2016

In June 2015 after a one-month gap a further two ICU patients were identified carrying *C. auris* (Fig. 1). The possibility of a healthcare associated transmission within the ICU was raised and prompted a review of possible routes of transmission including processes for washing patients, shared equipment, ventilation, lapses in cleaning and hand hygiene. Environmental sampling of the clinical area surrounding colonized patients demonstrated contamination with *C. auris* of horizontal surfaces such as the floor around bed sites, trollies, radiators, windowsills, equipment monitors and key pads, and also one air sample. Further attention was focused on infection prevention and control (IPC) measures. Prospective surveillance of *C. auris* from clinical specimens and screening of *C. auris* positive patient contacts was also introduced to actively identify further transmissions. No further cases occurred over a three-month period until the end of September 2015 when a series of new cases occurred, and by November 2015 there were a total of nine *C. auris* cases including several with candidaemia. Formal outbreak meetings were set up with regular review and audit of IPC measures (described below). The outbreak slowed down over the Christmas period but resurged in January 2016 with a steep increase of cases, reaching a total of 50 cases over a 16 months period by July 2016 (Fig. 1). Due to the movement of *C. auris* positive patients and their contacts from the ICU to other wards, new cases also occurred briefly in those wards.

### Infection control and prevention measures

A formal outbreak investigation led to the implementation of enhanced measures to limit transmission including isolation of all positive *C. auris* patients, cohorting of their direct patient contacts as well as ceasing new admissions to the affected rooms. All positive patients were kept under strict isolation for the duration of their hospital stay. All direct contact patients were screened for the presence of this yeast in sites including nose, axilla, groin, throat, rectum or faeces, vascular line exit sites and clinical samples such as urine, wounds, drains and respiratory specimens. Route cause analysis revealed that the minimum contact period with a positive case or a contaminated environment for the acquisition of *C. auris* was ≥4 h. However, no single point source of transmission was identifiable. Direct contact patients were only de-isolated after three consecutive negative *C. auris* screens and screened weekly thereafter until discharge. The latter was introduced as one patient became positive after three consecutive negative screens.

As healthcare workers (HCW) have been implemented in the transmission of other *Candida* species in the past we have undertaken an extensive staff screening programme involving doctors, nurses, physiotherapists, catering and cleaning staff, dieticians, a Chaplin and ward administrators. Staff hands (agar impression plates), nose, axilla, groin and throat swabs were analysed for the presence of *Candida* [9]. Only one out of 258 HCW screened were found to have a *C. auris* positive nose swab (all other samples were negative). This nurse had been caring for a heavily *C. auris* colonized patient. After a five day decolonization protocol with chlorhexidine washes, nasal ointment and oral nystatin medication (as described below) repeat microbiology samples were negative suggesting transient carriage only. Retrospectively the staff member reported a skin allergy to alcohol gel which may explain suboptimal hand decontamination and subsequent colonization of the nose. However, as this HCW was not nursing any other previous or subsequent new *C. auris* cases it was felt that the outbreak was unlikely to be related to this person.

Strict contact precautions were introduced for all healthcare workers, cleaners and visitors on entering rooms where patients were isolated. These included the wearing of cuffed long-sleeved disposable gowns, gloves and aprons similar to Public Health England recommendations for carbapenemase producing enterobacteriaceae control [10]. For decolonization of *C. auris,* patients were prescribed twice daily 2 % chlorhexidine gluconate washes using single use wipes (Sage, Geneva, Switzerland) or aqueous 4 % chlorhexidine formulation, mouthwashing containing 0.2 % chlorhexidine (Corsodyl, GlaxoSmithKline, Brentford, UK) or chlorhexidine 1 % dental gel (Corsodyl) for patients on ventilator support and oral nystatin if oropharyngeal colonization was present. We also introduced the use of chlorhexidine impregnated protective disks for all central vascular catheter exit sites (BioPatch, Johnson & Johnson, Somerville, NJ, USA) to reduce line associated *C. auris* blood stream infections.

For environmental decontamination we implemented extreme measure for cleaning and disinfection of the patient rooms and equipment using 1000 ppm chlorine based products (Chlorclean, Guest Medical, Ashford, UK) three times a day. On discharge or transfer of a *C. auris* positive case the room was subjected to a terminal cleaning with 10,000 ppm chlorine based detergent (Haztab, Guest Medical) and any cleaned equipment was left in the room to be disinfected with hydrogen peroxide vapour (Bioquell Ltd, Andover, UK).

### Characteristics and typing of outbreak strains

*C. auris* appeared as beige coloured colonies on Chromogenic agar and species was confirmed using MALDI-TOF analysis. All isolates expressed high level fluconazole resistance (MIC: >256 mg/L) but the majority were susceptible to echinocandins (MIC 0.06-0.25 mg/L), 5-flucytosine (MIC <0.06-0.12 mg/L) with variable susceptibility to amphotericin B (0.5-2 mg/L). A small selection of isolates were tested for nystatin and terbinafine (1 mg/l) susceptibility.

**Fig. 2:**
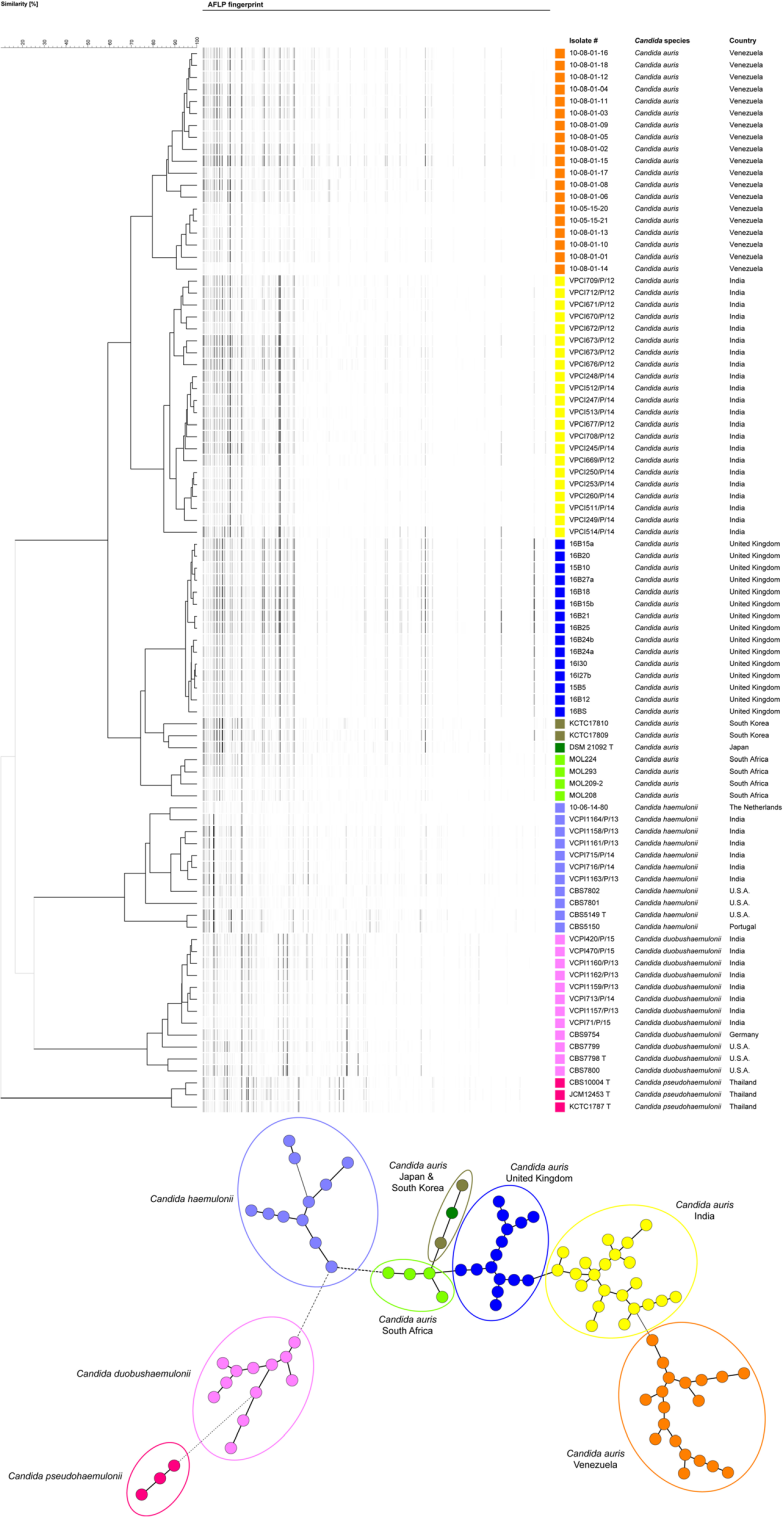
AFLP typing and of *C. auris*. UPGMA dendrogram of AFLP fingerprint analysis and an AFLP-derived minimum spanning tree of *C. auris* isolates from the UK (*n* = 15) compared to those from India (*n* = 22), Japan (*n* = 1), South Africa (*n* = 4), South Korea (*n* = 2), and Venezuela (*n* = 19). Isolates from the closely related sibling species *C. haemulonii* (*n* = 11), *C. duobushaemulonii* (*n* = 12) and *C. pseudohaemulonii* (*n* = 3) were included to serve as an outgroup. Cluster analysis showed that all species form distinct clusters based on the AFLP fingerprint profiles, demarcated by the black dendrogram lines or in the minimum spanning tree where branch lengths indicates the similarity between isolates with thick solid lines (up to 14.96), thin solid line (up to 29.25), thick dashed lines (up to 43.54), thin dashed lines (up to 57.83) and thin dotted lines (above 57.83). *C. auris* isolates that came from the same geographic region clustered together

AFLP has previously been shown to reliably distinguish *C. auris* from closely related species *C. haemulonii*, *C. pseudohaemuloni* and *C. duobushaemulonii*, and was performed on available outbreak isolates to identify a geographic origin of this outbreak [7, 8]. AFLP analysis of a selection of UK isolates (*n* = 15) was done and compared to isolates from India (*n* = 22), Japan (*n* = 1), South Africa (*n* = 4), South Korea (*n* = 2) and Venezuela (*n* = 19) using previously published methodology [7, 11]. The resulting dendrogram suggests that the London isolates form a distinct cluster compared to other global isolates (Fig. 2). The high degree of relatedness within the AFLP dendrogram (Fig. 2) suggests a single introduction of the infecting genotype into the hospital however analysis of whole-genome sequences is ongoing to confirm this preliminary observation.

### Clinical impact of *C. auris* and prevalence

In the majority of cases *C. auris* was confined to colonisation of skin sites or mucosa. However, a total of 44 % (*n* = 22/50) of patients required anti-fungal therapy with an echinocandin, amphotericin B and/or 5-flucytosine for possible or proven *C. auris* infection including nine episodes of candidaemia in a total of eight patients (Table 1). Some cases developed candidaemia despite the use of echinocandins which also did not reduce skin colonization. An independent mortality review demonstrated that there were no deaths directly attributable to infections by *C. auris*.

**Table 1:** Clinical manifestations of *C. auris* in patients

Clinical manifestation of *C. auris* cases	Percent (total number)
Colonization only	56 % (*n* = 28/50)
Candidaemia episodes (one patient had two episodes)	18 % (*n* = 9/50)
Possible sternal wound infection (culture positive and clinical signs of infection)	6.3 % (*n* = 3/50)
Possible urinary catheter infection (culture positive before and after catheter change and response to antifungal treatment)	2 % (*n* = 1/50)
Possible vascular line tip infection (positive line tip culture treated empirically with antifungal agent)	14 % (*n* = 7/50)
Presumed invasive candidiasis of unknown focus of infection	4 % (*n* = 2*/50)

*one patient had a raised BDG of 303 pg/mL (normal range <60 pg/mL)

In order to establish whether patients already carrying *C. auris* on admission to the hospital have contributed to the positive case load we analysed a random set of *C. auris* admission screens obtained between July 2015 and July 2016. The prevalence of *C. auris* in our admitted patient population was 0.04 % (*n* = 1/2246 screened patients).

## Discussion

*C. auris* is a globally emerging multidrug resistant fungal pathogen with the first clinical case being described in 2009 causing an ear infection in a Japanese patient [1]. Whilst isolated sporadic cases occur, there is a growing concern regarding the propensity of *C. auris* to cause widespread nosocomial outbreaks [2]. Several clusters have emerged globally including countries such as South Korea [3, 12], India [7, 8, 11, 13], South Africa [4], Pakistan [14], and hospitals in Latin America [5, 14]. Whole genome sequencing (WGS) demonstrated highly related *C. auris* isolates in the same geographic areas [14, 15]. Alarmingly, our outbreak is the first description of hospital acquired transmission leading to a large outbreak in a European country adding to the evidence that this multidrug resistant pathogen is capable of transmission in the health care setting causing potentially serious infections of a global concern. As carriage was negligible (0.04 %) in our admitted population, the observed rate of infection within our facility was 44 % with an 18 % rate of candidaemia amongst colonized patients. These values are high and pose a serious risk to critically ill patients. Unsurprisingly, intensive care stay has been reported as a major risk factor for *C. auris* infections in a recent case series from developing countries [5, 11, 13, 16]. The occurrence of candidaemia that is attributed to *C auris* appears increasingly common and is associated with mortality of up to 50 % in some countries, although not seen in our series or others [3–5]. The antifungal drug resistance is of particularly concern as it characteristically demonstrates high level resistance to azoles (particularly fluconazole) and in other studies has been shown to be multidrug resistant, including echinocandins and amphotericin [2].

Early identification of *Candida* species as recommended by the British Society for Medical Mycology best practice is not only important for the appropriate use of antifungal treatment but also in order to implement effective infection control measures [17]. Many microbiology laboratories currently do not routinely speciate non-*Candida albicans* isolates or utilize yeast identification methods such as chromogenic agar, biochemical tests (API) or automated systems such as VITEK which do not speciate this pathogen or may misidentify *C. auris* as yeasts such as *Candida haemulonii*, *Candida sake*, and *Rhodotorula mucilaginosa* [6, 11]. Currently, reliable methods for speciation are molecular based methods such as PCR, AFLP fingerprinting, sequencing analysis, and MALDI-TOF biotyping [6–8].

Our data shows an innate resilience of this fungal pathogen for survival and persistence in the clinical environment, the ability for rapid colonization of patient’s skin and high transmissibility within the health care setting leading to a serious and prolonged outbreak. The management of this outbreak has been a costly challenge despite extremely stringent IPC measures focussing particularly on rapid identification of carriers of *C. auris*, prompt isolation and decolonisation of positive patients, extensive contact tracing and screening of patients, and enhanced cleaning and decontamination of the environment including medical equipment. We learned that despite daily chlorhexidine washes as recommended for the prevention of healthcare associated infections, patients continue to be colonized [18]. This is presumably due to reinfection from within their bedding and clothing although a reduced susceptibility of *C. auris* to chlorhexidine may be a possibility which we are currently investigating.

Our results from environmental screening demonstrate that positive patients can shed *C. auris* into the close environment posing a risk of continuous transmission. Although we have not been able to identify a specific point source we believe the prolonged outbreak is likely to be due to low level environmental contamination. Despite a comprehensive review of modern technologies for environmental decontamination there is currently no published data in the literature on the effectiveness of cleaning agents or decontamination of the environment for *C. auris* specifically [19]. Nevertheless, from our own practical experience based on environmental screening pre-and post-cleaning we have now reasonable confidence that high strength chlorine based agents and hydrogen peroxide vaporisation are effective. This highlights the importance of strict adherence to cleaning/decontamination protocols and the need for isolating/cohorting all positive patients.

## Conclusion

Based on our experience we would recommend, firstly, being vigilant in searching for *C. auris* in clinically significant specimens in high risk hospital environment/high risk patients such as intensive care units. There should be a high level of suspicion when isolating non-*C. albicans* isolates with fluconazole resistance.

Secondly, it is advisable to implement stringent IPC measures for all positive *C. auris* cases including strict isolation/cohorting and decolonization using chlorhexidine and oral nystatin (if susceptible) and to perform extensive screening of all direct contacts. More information is needed on effective skin decolonisation regimens in order to prevent invasive infections and further shedding of this yeast into the environment.

Thirdly, regular environmental and equipment cleaning with high strength chlorine-based agents and possibly hydrogen peroxide vaporization is key to the reduction of *C. auris* in the environment. In our experience, once the yeast has been introduced in the environment it poses a risk of transmission to patients and is difficult and costly to eradicate. Regular auditing of IPC practices in particularly compliance with hand washing/decontamination should be undertaken. Although in our investigation we have not been able to detect *C. auris* carriage on HCW hands but others have shown that hands can be key vectors in the transmission of *Candida* species either via direct contact with colonised/infected patients or indirect contact with contaminated environment or equipment [9].

All in all, because there is very little known about the factors promoting environmental resilience and transmission, the mechanisms of resistance to antifungal drugs or disinfectants and properties contributing to prolonged host colonization, the management of outbreaks in healthcare facilities will remain a difficult challenge.

## Abbreviations

AFLP: Amplified fragment length polymorphism
BC: Blood culture
BDG: β-D-glucan
ICU: Intensive care unit
IPC: Infection prevention and control
MALDI-TOF: Matrix Assisted Laser Desorption Ionization-Time of Flight mass spectrometry
MIC: Minimal inhibitory concentration
PCR: Polymerase chain reaction
UTI: Urinary tract infection
